# Insights from the analysis of alginate lyase protein model from Pseudomonas fluorescens towards the understanding of mucoid biofilm disruption

**DOI:** 10.6026/97320630013318

**Published:** 2017-09-30

**Authors:** Gurjant Singh, Mahesh Kulharia

**Affiliations:** 1 Centre for Human Genetics and Molecular Medicine, School of Health Sciences, Central University of Punjab, Bathinda 151001, India; 2 Centre for Computational Sciences, School of Basic and Applied Sciences, Central University of Punjab, Bathinda 151001, India

**Keywords:** Bacterial biofilms, alginate lyase, alginate, Pseudomonas fluorescens, GROMACS, molecular dynamics, homology modeling

## Abstract

Bacterial biofilm is a protective, slippery and slimy coat secreted by bacterial cells. It helps in attaching to moisturized surfaces during colonization. Alginate is an important component as it is essential for retention of water and nutrients in biofilms. It is a polysaccharide consisting of β-D-mannuronic acid (M) and α-L-guluronic acid (G) monomers with 1-4 linkage. The alginate lyase (AlgL) secreted by certain bacteria is capable of degrading alginate into oligo-uronides by β-elimination of the glycosidic bond. Therefore, it is of interest to analyze the simulated (GROMACS force filed) structure protein model (homology based on template 4OZV) of AlgL from Pseudomonas fluorescens to gain functional insight mucoid biofilm disruption. We report root mean square deviation (RMSD) and radius of gyration (Rg) profiles of the simulated (molecular dynamics) AlgL protein homology model in this context towards biofilm discruption.

## Background

Bacterial biofilm is a slippery and slimy coat secreted by bacterial cells. This allows them to get attached to moisturized-surfaces to get colonized together to survive and grow in the environment. The biofilm grow thick and are often seen by naked eyes. This is the mode of growth and protection used by bacteria and it is observed in the environment [1]. Bacterial biofilms are generally resistant to most of the antimicrobial factors [2, 3] and microbes under bio-films become resistant to the host immune system and are usually less susceptible to various antibiotics [4]. Different bacteria secrete various forms of biofilms. These include grampositive bacteria such as Staphylococcus sp., and various lactic acid bacteria [5]. It also includes gram-negative bacteria such as Escherichia coli and Pseudomonas sp. as well as different nitrogenfixing bacteria such as Sinorhizobium meliloti and Rhizobium leguminosarum etc. [6].

Alginate is an important polymer found in mucoid biofilms. Alginate does not have a significant role in the synthesis and maintenance of non-mucoid biofilms [7]. However, it has an important role in the formation and maintenance of the mucoid biofilms. It is needed for the retention of water and nutrients in biofilms [8]. Alginate polymer is composed of monomers, β-Dmannuronic acid (M) and α-L-guluronic acid (G). G is C5 epimer of M. This is secreted by brown-seaweeds and bacterial species (Pseudomonas sp. and Azobacter etc.) [9]. It is an important component of mucoid bacterial biofilms and it attracts attention.

Alginate lyase (AlgL) is one of the proteins (factors) essential for the biosynthesis of alginate [10] and AlgL is also able to degrade the alginate polymer into oligo-uronides. AlgL digests alginate by β-elimination of the glycosidic bond [11]. It yields various unsaturated oligosaccharides and monomers of uronic acid from alginate. This finds applications in different areas. Different kinds of AlgLs are found in various algae, soil microorganisms, marine microorganisms and marine invertebrates etc. AlgLs can be classified as polyM specific, polyG specific and polyMG specific lyases based on their specificity toward substrates. AlgLs also have endo and exo degradation activities according to their specificity [12]. Nonetheless, the three dimensional structure of AlgL is not known. Therefore, it is of interest to analyze the simulated (GROMACS force filed) structure protein model (homology based on template 4OZV) of AlgL from Pseudomonas fluorescens to gain functional insight mucoid biofilm disruption. We report root mean square deviation (RMSD) and radius of gyration (Rg) profiles of the simulated (molecular dynamics) AlgL protein homology model in this context to gain molecular insights.

## Methodology

### Query sequence selection

The database UniProtKB was used to select a query sequence with unknown structure. UniProtKB-Q3KHR0 entry was selected from UniProtKB. FASTA format sequence was downloaded from UniProtKB. This was further refined using the National Center for Biotechnology Information (NCBI) search interface and the sequence with sequence ID# 011332553.1 was selected for this study.

### Selection of template protein structures

Basic local alignment search tool (BLAST) for proteins was used to find template structures for model building against nonredundant PDB as a search database. The protein structure with maximum identity (PDB ID: 4OZV_A with 65% identity) was selected as the template structure.

### Protein structure modeling using Modeller9.16

Modeller9.16 was used to predict the structure of the query sequence. A laptop with Intel(R) Pentium(R) @1.99GHz processor and Microsoft Windows 7 Ultimate OS was used for this purpose. Query sequence was stored in PIR format. Modeller was used to align the query sequence in file qseq.ali with the template structure in the pdb file. This produced five models with the different Discrete Optimized Protein Energy (DOPE) score and GA341 score. Model #2 was selected as best model because it had lowest DOPE score and full GA341 score.

### Simulation of model using GROMACS

The simulations were done with GROMACS program on a laptop with Linux (Ubuntu 14.04 LTS) OS. The process of simulation consisted of topology generation, defining box and solvation, charge neutralization, energy minimization, equilibration, timedependent observation and analysis.

### Qualitative Model Energy ANalysis (QMEAN) Analysis

QMEAN server checked the quality of the protein structure model. The QMEAN score of the model is 0.61 in this case.

## Results & Discussion

**Figure 1:**
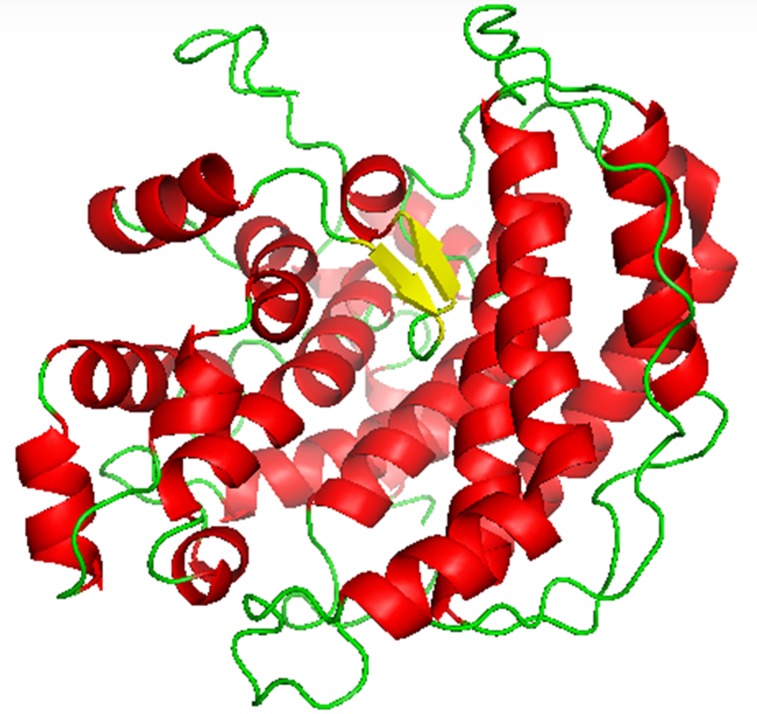
A pymol view of the model is shown. The structure model of AlgL consists of 18 alpha helices of various lengths, 2 beta sheets (both 3 residues long), and 20 loops of various lengths is illustrated. The alpha helices are shown in red, beta sheets are shown in yellow and loops (random coils) are shown in green.

**Figure 2:**
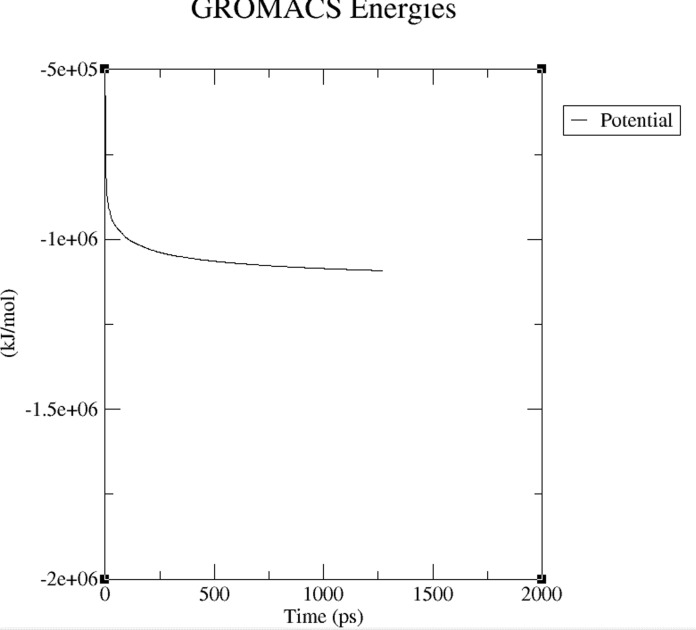
A potential energy minimization plot is shown and it is reduced to its lowest possible level.

**Figure 3:**
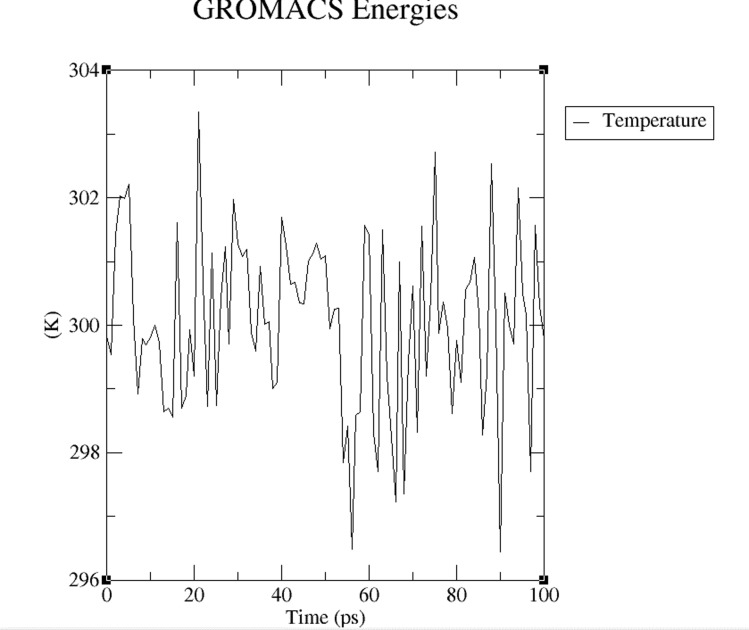
A temperature progression plot is shown. The temperature was stabilized at 300K over a time period of 100 ps. The temperature of the system is fluctuating but the average value of temperature is stabilized at 300K.

**Figure 4:**
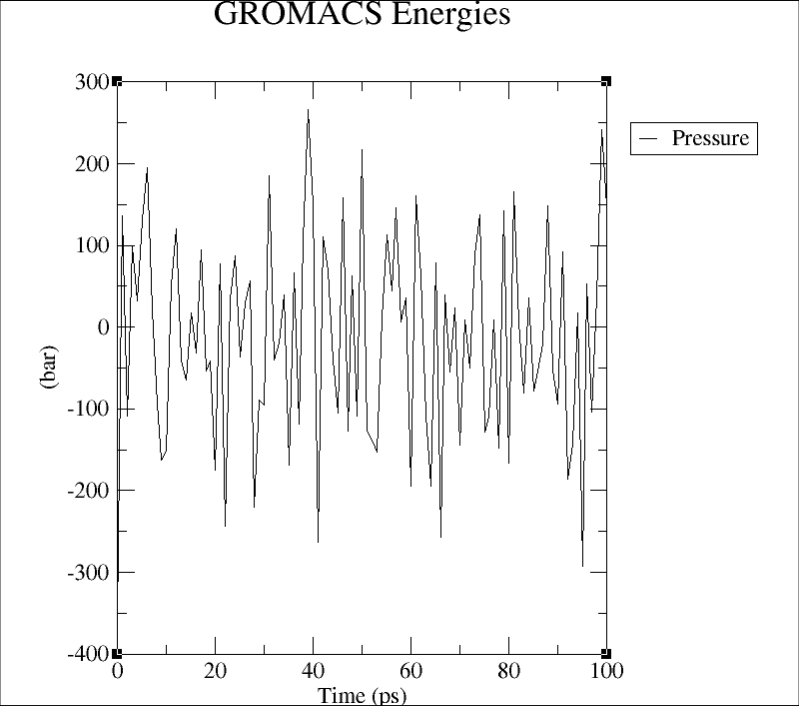
A pressure progression plot is shown. The pressure of the system fluctuated (about 100 bars per ps) as expected and its average value of pressure over the course becomes zero.

**Figure 5:**
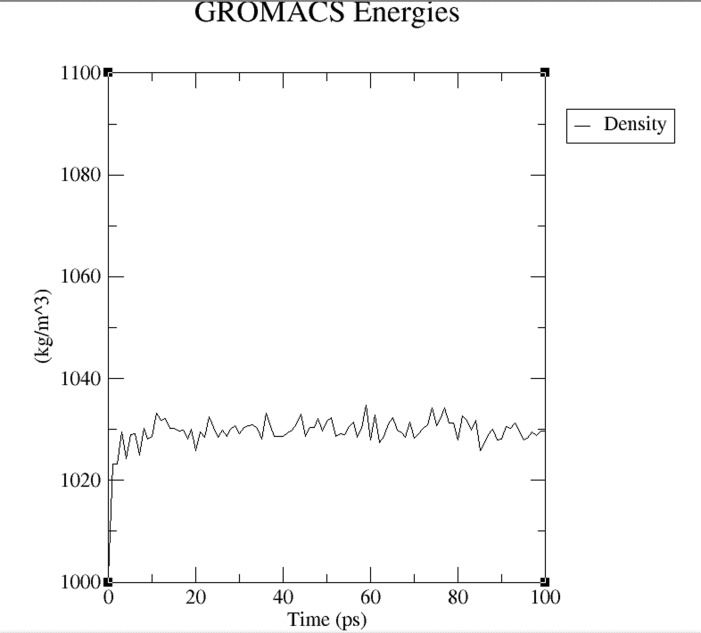
A density stabilization plot is shown. The density stabilization plot over time converges to an average value.

The structure model of AlgL consists of 18 alpha helices of various lengths, 2 beta sheets (both 3 residues long), and 20 loops of different lengths as shown in Figure 1. The alpha helices are shown as red, beta sheets are yellow and loop or random coils are shown green in Figure 1. The Ramachandran plot of the model was generated using the UCLA-DOE LAB server. Most residues do not lie in the disallowed regions of the plot. The potential energy minimization of the system is shown in Figure 2 and the temperature progression (equilibration) is shown in Figure 3. It is shown that the temperature reached 300K (target point) in the process and remained stable. Equilibration the pressure (Figure 4) and density (Figure 5) stabilization was completed subsequently. The system pressure fluctuated (about 100 bars per ps) as expected. However, the average value of pressure becomes zero during simulation as shown in Figure 4.

**Figure 6:**
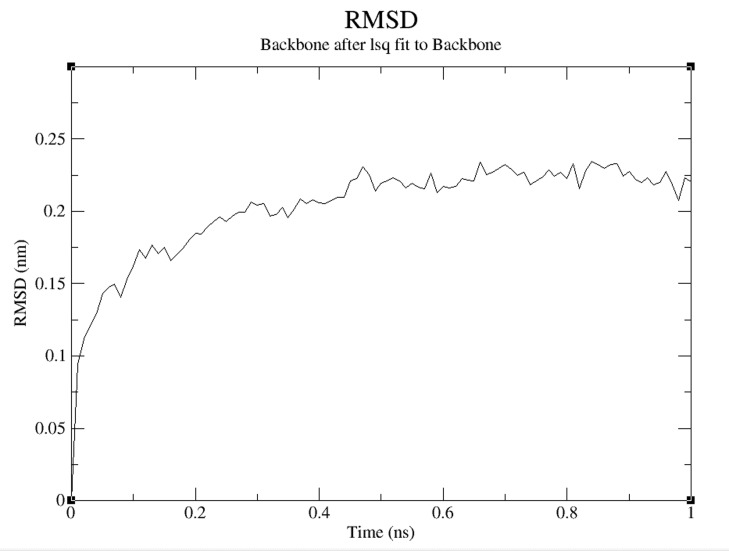
A RMSD plot is shown over time. This is relative to the energy minimized template structure.

**Figure 7:**
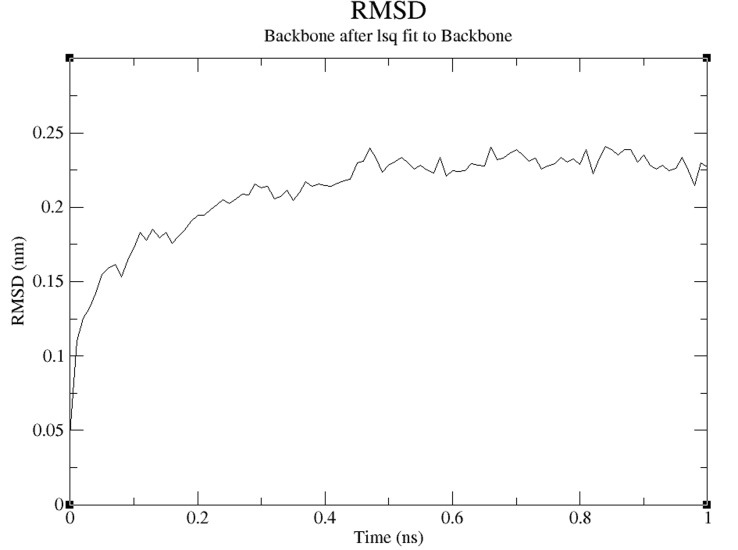
A RMSD (with reference to crystal structure) plot is shown.

**Figure 8:**
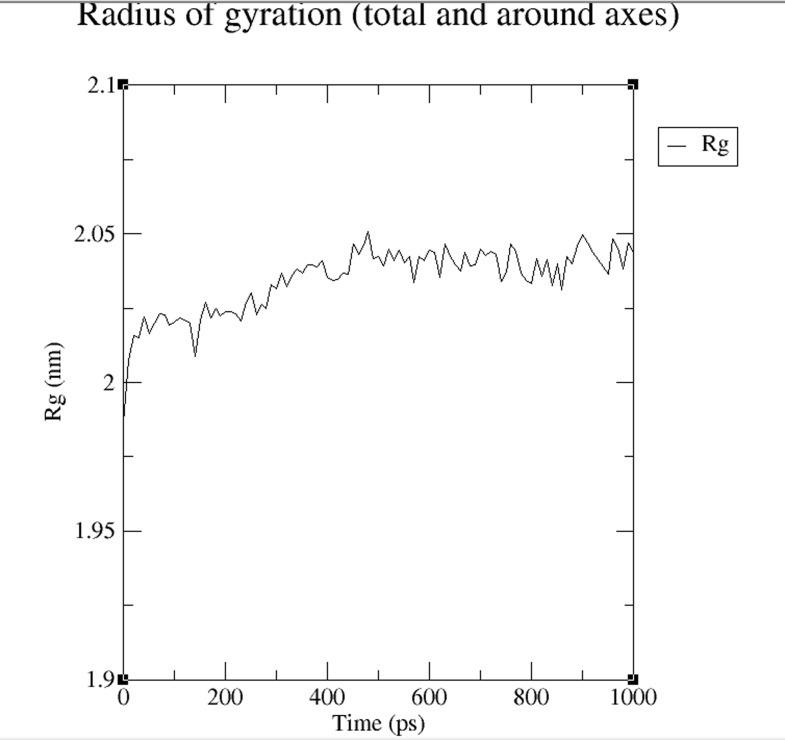
A radius of gyration (Rg) plot is shown. The plot illustrates that the Rg value of protein structure model at a temperature about 300K remained reasonably invariant and hence stable.

The density stabilization plot over time stabilized to an average value as shown in Figure 5. The Root Mean Square Deviation (RMSD) relative to a structure, which is present in minimized, equilibrated system is shown in Figure 6. The RMSD relative to crystal structure is also shown in Figure 7. Both of the plots show RMSD levels of about 0.225 with a stable structure. The Radius of gyration (Rg) of the structure is shown in Figure 8. The plot in Figure 8 illustrates that the Rg value of protein structure model at a temperature of about 300K remained reasonably invariant. This means the protein structure model is structurally stable at 300K.

**Figure 9:**
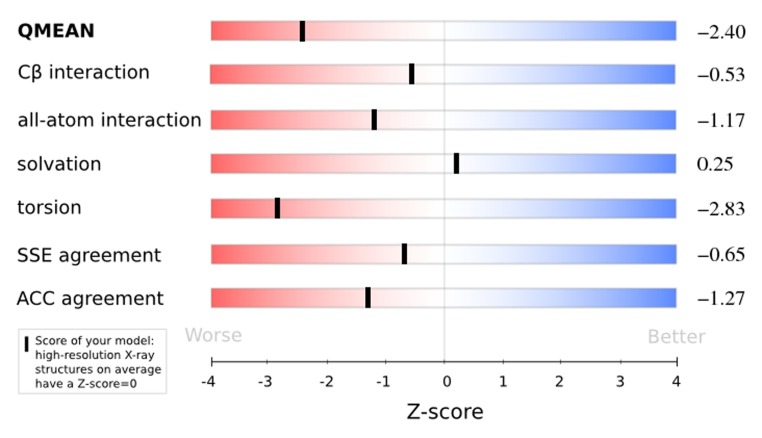
Z-score of simulated structure model according to QMEAN scoring functions is shown. The normalized QMEAN score of simulated protein structure model is 0.57.

**Figure 10:**
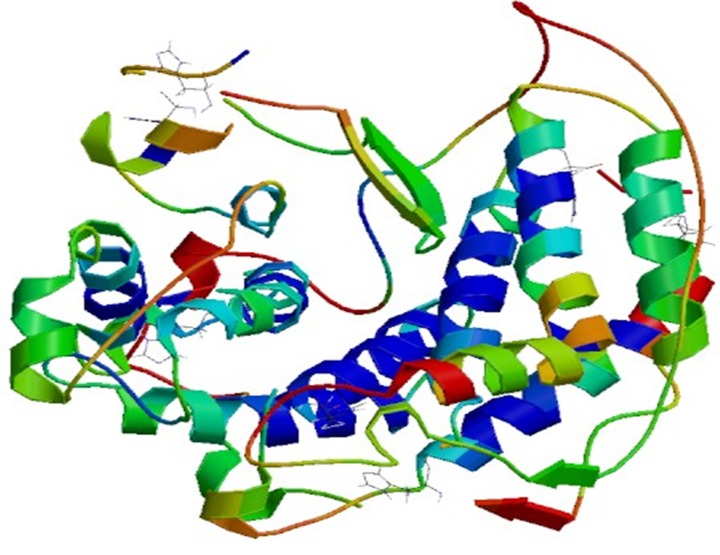
Residue errors in the simulated model are shown. It ranges from less than 1Å (reliable regions, shown blue) to above 3.5Å (unreliable regions, shown red). The others in between regions are shown green to yellow.

The z-score of simulated protein structure model according to different scoring functions of QMEAN is shown in Figure 9. The residue error calculated by QMEAN server is shown in Figure 10. It ranges from less than 1 Å (reliable regions, shown blue in Figure 10) to above 3.5 Å (unreliable regions, shown red in Figure 10). The other regions are shown green to yellow in Figure 10.

The density plot (over the QMEAN score) for all the reference models used in the calculation of Z score was also plotted. The position of the model was observed with QMEAN score = 0.57 and Z score = 2.40. The Ramachandran plot of the simulated model did not show any non-Gly residues in the disallowed regions of the plot suggesting acceptable model quality. These data provide information in the understanding of AlgL from P. fluorescens towards its molecular function.

## Conclusion

The homology structural model of AlgL from P. fluorescens is described. The model of AlgL is rich in alpha helices and random coils. It consists of 18 alpha helices of various lengths, 2 beta sheets (both 3 residues long), and 20 loops of various lengths. It is of interest to analyze the simulated (GROMACS force filed) structure protein model (homology based on template 4OZV) of AlgL from Pseudomonas fluorescens to gain functional insight mucoid biofilm disruption. We report root mean square deviation (RMSD) and radius of gyration (Rg) of the simulated AlgL model in this context to gain molecular insights towards biofilm disruption.
